# Silver-Composited Polydopamine Nanoparticles: Antibacterial and Antioxidant Potential in Nanocomposite Hydrogels

**DOI:** 10.3390/gels9030183

**Published:** 2023-02-27

**Authors:** Geun-Jin Song, Yeon-Su Choi, Hee-Sook Hwang, Chung-Sung Lee

**Affiliations:** 1 Department of Pharmaceutical Engineering and Biotechnology, Sun Moon University, Asan 31460, Republic of Korea; 2 Department of Pharmaceutical Science, University of California, Irvine, CA 92697, USA; 3 Department of Pharmaceutical Engineering, Dankook University, Cheonan 31116, Republic of Korea

**Keywords:** silver nanoparticle, antibacterial activity, antioxidant activity, hydrogel, dopamine

## Abstract

(1) Background: Infections of pathogenic microorganisms can be life-threatening due to delayed healing or even worsening conditions in tissue engineering and regenerative medicine. The excessive presence of reactive oxygen species in damaged and infected tissues causes a negative inflammatory response, resulting in failed healing. Thus, the development of hydrogels with antibacterial and antioxidant abilities for the treatment of infectious tissues is in high demand. (2) Methods: We herein describe the development of green-synthesized silver-composited polydopamine nanoparticles (AgNPs), which are fabricated by the self-assembly of dopamine as a reducing and antioxidant agent in the presence of silver ions. (3) Results: The facile and green-synthesized AgNPs have a nanoscale diameter with mostly spherical shapes, with various shapes coexisting. The particles are stable in an aqueous solution for up to 4 weeks. In addition, remarkable antibacterial activity against Gram-positive and -negative bacterial strains and antioxidant capabilities were evaluated by in vitro assays. When incorporated into biomaterial hydrogels at concentrations above 2 mg L^−1^, the hydrogels produced powerful antibacterial effects. (4) Conclusions: This study describes a biocompatible hydrogel with antibacterial and antioxidant activities from the introduction of facile and green-synthesized AgNPs as a safer tool for the treatment of damaged tissues.

## 1. Introduction

Hydrogels are frequently considered medical options in tissue engineering and regenerative medicine [1,2,3,4]. They support a damaged and injured site and can deliver a variety of therapeutic or bioactive agents, including drugs and cells [5,6]. The hydrogel can be easily applied by injection to irregular three-dimensional areas or utilized to protect the damaged site from pathogenic infection [7]. However, hydrogels are highly biocompatible, so they can be vulnerable to bacterial pathogens due to the very moist microenvironment of the hydrogels [8,9]. In particular, hydrogels for tissue regeneration can be inhibited by bacterial infection, which can cause significant adverse events resulting in treatment failure. Thus, controlling bacterial infection in hydrogels can be important to achieve a proper therapeutic outcome in the use of hydrogels for tissue engineering and regenerative medicine.

Monovalent silver ions are a well-known antibacterial agent that have a strong oligodynamic effect [10]. The attention to silver-based agents as alternative antibacterial agents against a wide range of microorganisms has developed because the use of antibiotics can cause bacterial resistance. The silver ions can be formed into a nanosized colloidal suspension resulting in the generation of silver nanoparticles (AgNPs) [11]. The antibacterial activity of AgNPs is more effective than that of monovalent silver ions due to their electronic effects. With strong antibacterial activity, AgNPs have been used for effective regeneration and treatment in biomedical applications [12,13,14]. AgNPs have been fabricated using various techniques, including chemical reduction, gamma irradiation, microemulsion, electrochemical procedures, microwaves, and laser ablation [15,16,17]. Although these techniques produce AgNPs with good yields, there are limitations such as the use of toxic chemicals, high operating costs, and high energy requirements that need to be addressed [16,18]. Given the shortcomings of these techniques, cost-effective and energy-efficient alternatives have been considered for the synthesis of AgNP using eco-friendly approaches (called green synthesis) involving microorganisms, plant extracts, and natural polymers as reducing and stabilizing agents.

The green synthesis of AgNPs has been developed using plant extracts and biomaterials. Dopamine has a unique property represented by mussel-inspired chemistry, and it has received a great deal of attention in the fields of biology and medicine [19]. Dopamine can easily polymerize under alkaline conditions through self-polymerization, resulting in the fabrication of polydopamine nanospheres [20,21,22,23]. In particular, catechol groups of dopamine can provide an active surface for reducing and absorbing metal ions and act as powerful reactive oxygen species (ROS) scavengers [24]. Thus, polydopamine nanospheres are generally considered safe biomaterials with excellent biocompatibility and biological properties.

**Scheme 1:**
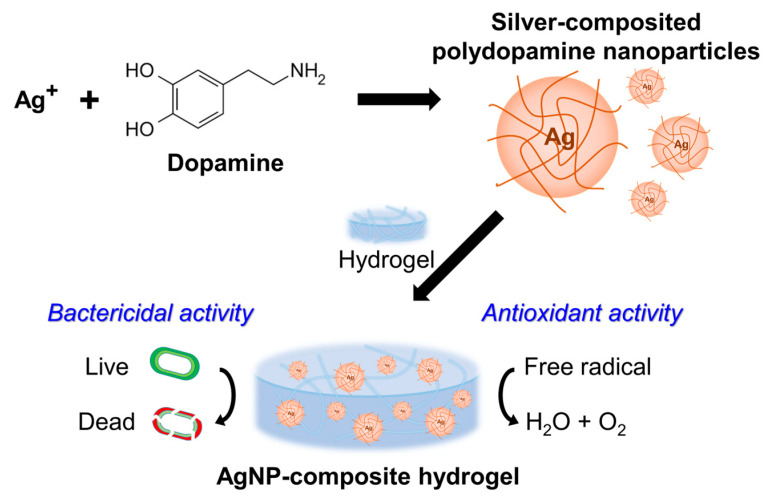
Schematic illustration of AgNP-composite hydrogel with facile and green-synthesized AgNPs with dopamine.

Herein, we present a facile and green synthesis of AgNP with dopamine. There is no need for additional reducing agents in this synthetic procedure (Scheme 1). Additionally, the synthesized AgNPs are incorporated into the biomaterial hydrogels to generate a hydrogel that can be widely applied in the field of tissue engineering. The characterization of AgNPs was performed using dynamic light scattering (DLS), observation through electronic microscopy, and UV–visible absorbance. Subsequently, the antibacterial activity against Gram-positive and -negative bacteria of free AgNPs and AgNP-incorporated hydrogels, the antioxidant activity for scavenging of ROS, and biocompatibility against bone marrow stromal cells (BMSCs) were evaluated in vitro. We chose guanidylated chitosan and carboxymethyl cellulose (GC/CMC) as well as gelatin methacryloyl (GelMA) hydrogels, which have different hydrogel-forming mechanisms (electrostatic interaction and photocrosslinking, respectively). These two types of hydrogels were used as model hydrogels.

## 2. Results and Discussion

### 2.1. Preparation and Characterization of AgNPs

**Figure 1:**
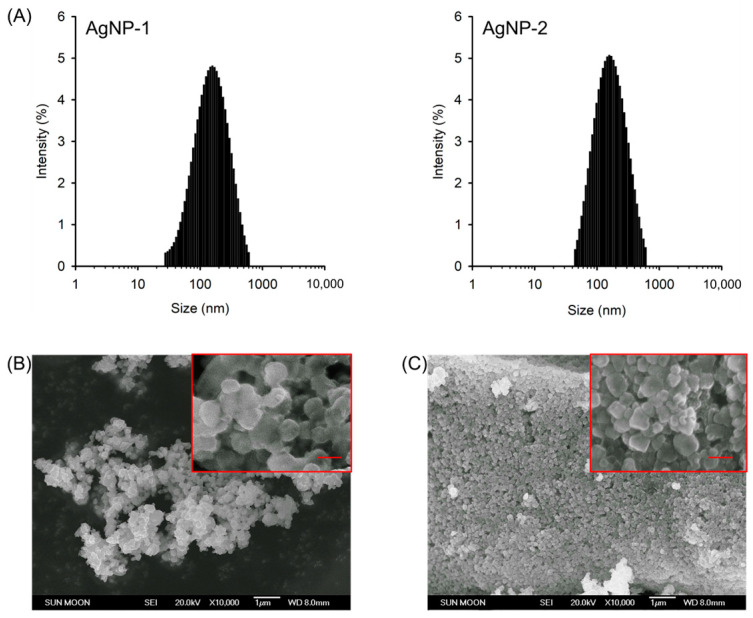
Physicochemical characterizations of AgNPs. Representative size distributions of (**A**) AgNP-1 and AgNP-2. Representative SEM images with a low magnification (×10,000) of (**B**) AgNP-1 and (**C**) AgNP-2 (Inset: representative SEM images with a high magnification, ×100,000. Scale bars indicate 200 nm).

First, we synthesized AgNPs with dopamine as a reducing and stabilizing agent through the oxidation reaction of the catechol group and the reduction of silver ions. The reaction was performed in an alkaline solution with two different conditions, resulting in a synthesis of AgNP-1 (0.03 M dopamine and 0.06 M silver nitrate) and AgNP-2 (0.05 M dopamine and 0.10 M silver nitrate). The oxidation of catechol groups in dopamine in an alkaline solution generates nanoparticles by polymerization and self-assembly. In the presence of silver ions during this process, the silver ions can bind with the catechol groups of dopamine, resulting in metal coordination bonds between silver ions and catechol [25]. A series of physicochemical characterizations, including size, zeta potential, and morphology were determined using DLS measurements and SEM observations (Figure 1). The sizes of AgNP-1 and AgNP-2 were 147.6 ± 9.6 and 166.3 ± 2.7 nm with a narrow distribution (polydispersity index [PDI] < 0.2), respectively (Figure 1A). Additionally, the zeta potential measurements demonstrated a highly positive charge (22.1 ± 0.5 mV for AgNP-1; 40.6 ± 8.8 mV for AgNP-2), indicating that they were stable in a colloidal solution. SEM observation also showed that the nanoparticles had an irregular nanoscale morphology and a narrow distribution (Figure 1B,C).

**Figure 2:**
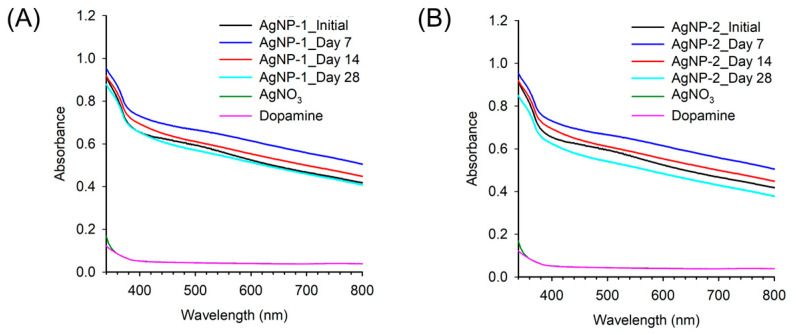
UV–vis absorbance spectra of (**A**) AgNP-1 and (**B**) AgNP-2 tracked for 28 days after being stored at 25 °C.

The absorption spectra of the AgNPs were measured (Figure 2). No specific surface plasmon of silver nanoparticles was shown by the samples because polydopamine has intrinsic absorbance in a broad range of wavelengths; whereas the spectra for dopamine or silver nitrate solution pre-reaction had no signal, so the absorbance signals of AgNPs were significantly increased, indicating successful nanoparticle production. Furthermore, no significant changes in the absorbance signal were observed over 28 days, indicating a stable colloid, which provides an advantage that could develop into a future translation for clinical use. AgNP-1 and AgNP-2 showed a difference in zeta potential values; a highly positive charge can cause cytotoxicity, even though there is no significant difference between the AgNPs in terms of physicochemical and optical properties. Based on these evaluations, subsequent evaluations were performed using AgNP-1, which has a comparatively lower zeta potential.

### 2.2. In Vitro Antibacterial Activity

**Figure 3:**
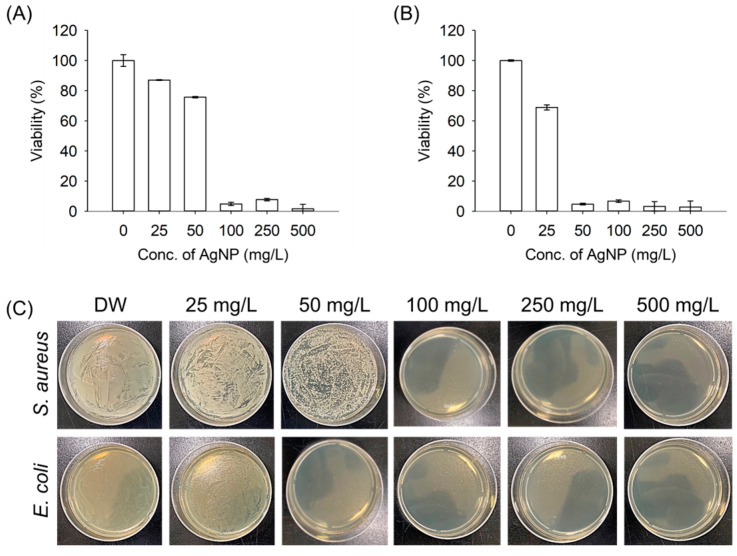
Antibacterial activity of AgNPs against bacterial strains. Quantitative antibacterial activity of AgNPs against (**A**) *S. aureus* and (**B**) *E. coli* (*n* = 3). (**C**) Photographs of bacterial strains incubated with different AgNP concentrations and then grown on agar media.

The antibacterial activity of the resulting AgNPs was determined against representative Gram-positive (*S. aureus*) and Gram-negative (*E. coli*) bacterial strains by evaluating the levels of minimum inhibitory concentration (MIC) using a broth dilution method and minimum bactericidal concentration (MBC) in the presence or absence of AgNPs at a range of concentrations, 0 to 500 mg L^−1^ (Figure 3) [26,27]. The AgNP in the MIC test showed effective suppression against *S. aureus* with MICs of approximately 65 mg L^−1^ and *E. coli* with an MIC of approximately 30 mg L^−1^ (Figure 3A,B). To assess MBC measurement, both bacterial strains were incubated with AgNP solutions at various concentrations and further grown on agar media (Figure 3C). The antibacterial activity significantly increased with the increasing concentration of AgNPs in comparison to the control distilled water (DW) group. The MBC, a concentration of complete bacterial killing, was observed at 100 or 50 mg L^−1^ against *S. aureus* and *E. coli*, respectively. These results indicate that the synthesized AgNP shows excellent antibacterial properties against broad-spectrum bacterial strains resulting in the complete killing of both Gram-positive and Gram-negative bacterial strains at low concentrations. In particular, the antibacterial activity of our AgNPs is higher than the commercially-available AgNP mentioned in the previous report [27]. The electrostatic interaction of positively charged AgNPs with negatively charged membranes of both bacterial strains could promote the rapid adsorption of nanoparticles on the bacterial surface leading to the destabilization of the membrane integrity. The results of a series of antibacterial assays revealed that the concentration of *E. coli* for effective bacterial activity was lower than that of *S. aureus*. These results can be reasonably understood based on the differences in the presence of the cellular walls of Gram-positive strains and Gram-negative strains.

**Figure 4:**
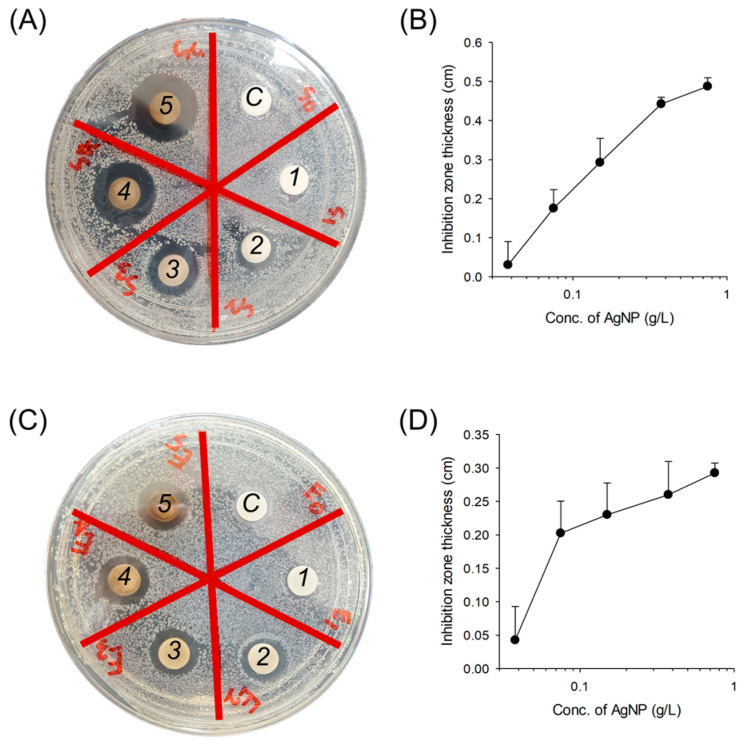
Antibacterial activity of AgNPs against bacterial strains. Antibacterial activity of AgNPs against (**A**) *S. aureus* and (**C**) *E. coli* determined by the disk diffusion method. The quantification of inhibition zone thickness for the result of disk diffusion test against (**B**) *S. aureus* and (**D**) *E. coli* (*n* = 3). C = control, distilled water. Areas 1–5 = AgNPs at concentrations of 38, 75, 150, 375, and 750 mg L^−1^ AgNPs, respectively.

We next performed further antibacterial assessments using the disk diffusion method (Figure 4). The result showed dose-dependent bactericidal activity against both Gram-positive (*S. aureus*) and Gram-negative (*E. coli*) strains, resulting in an increase in the inhibition zone thickness (Figure 4A,C). While the control groups had no inhibition zone, the concentration of all samples ranging from 38 to 750 mg L^−1^ clearly had inhibition zones. Although high-dose treatment showed an effective killing of both bacterial strains, the antibacterial activity against *E. coli* at a low dose, 38 mg L^−1^, was higher than that of *S. aureus*, which is similar to the results of predetermined antibacterial capacities (Figure 4B,D).

### 2.3. In Vitro Antioxidant Activity

Since the catechol group of dopamine is well known to have antioxidant activity, it is possible that the synthesized AgNPs in this study could scavenge free radicals and ROS that can be produced during the regeneration processes of damaged and injured tissues [24,28].

**Figure 5:**
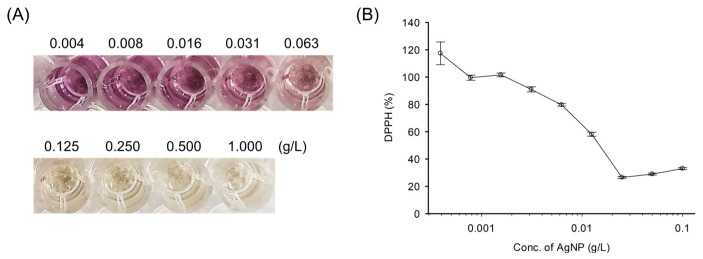
Antioxidant activity of AgNPs against DPPH radicals. (**A**) Photograph of DPPH radicals scavenging assay. (**B**) DPPH radical scavenging activities of AgNPs at various concentrations.

Toward this aim, the antioxidant activity was assessed using a 2,2-diphenyl-1-picrylhydrazyl (DPPH) radical scavenging assay (Figure 5). Antioxidants represent a form of opposite oxidants that prevent or delay cell damage by oxidants such as ROS and free radicals [29]. DPPH is one of the assays that determine antioxidant capacity [8]. The purple color of DPPH changes to a pale-yellow color when DPPH reacts with a hydrogen donor and is converted to a reduced form. As shown in Figure 5A, the level of purple color reduction depends on the antioxidant concentration. Since AgNPs scavenge ROS generation, the purple color changes to pale yellow which presents excellent antioxidant activity. The results clearly support the effective antioxidant potential of AgNPs. The antioxidant activity of AgNPs at a concentration of 31 mg L^−1^ resulted in a decrease in the purple color for DPPH radicals (Figure 5A). A concentration of more than 125 mg L^−1^ demonstrated near complete scavenging of DPPH radicals. The quantitation of remaining DPPH radicals was performed by measuring UV–visible absorbance at 570 nm (Figure 5B). The DPPH antioxidant activity for AgNP was gradually enhanced by increasing the concentration of AgNPs (9.0% for 31 mg L^−1^, 20.3% for 63 mg L^−1^, 41.9% for 125 mg L^−1^, and 73.5% for 250 mg L^−1^). Thus, this finding indicates that the synthesized AgNPs can scavenge ROS and that they have excellent antioxidant properties.

### 2.4. In Vitro Antibacterial Activity of AgNP-Incorporated Hydrogel Composites

**Figure 6:**
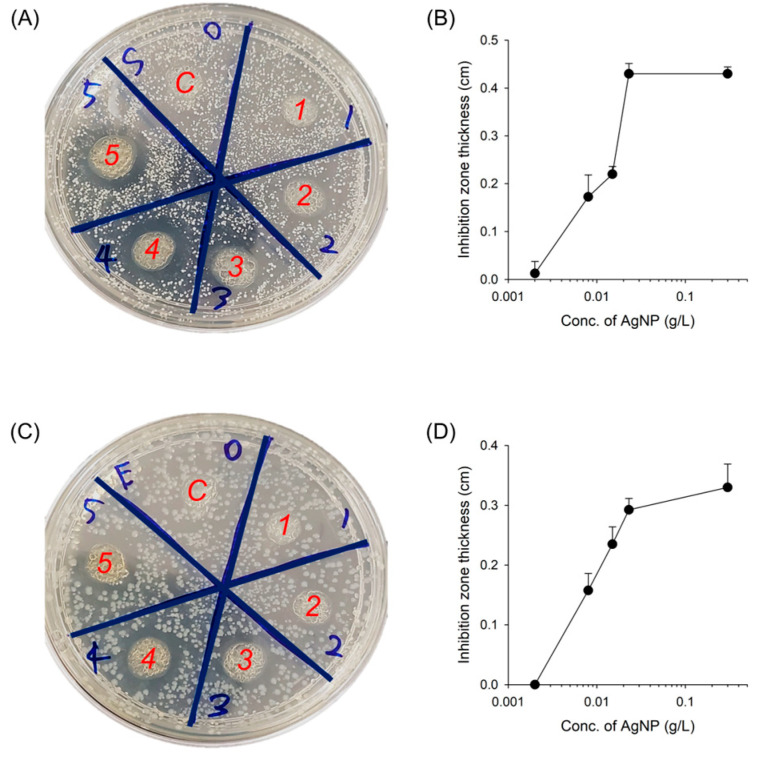
Bactericidal activity of AgNP-incorporated GC/CMC hydrogels. Representative image of bacterial colonies formed by (**A**) *S. aureus* and (**C**) *E. coli* with the AgNP-incorporated hydrogels placed on the agar petri dish for a day to analyze the inhibition zone. C = control, AgNP-free hydrogels. Areas 1–5 = AgNP-incorporated hydrogels at concentrations of 2, 8, 15, 23, and 30 mg L^−1^ AgNPs, respectively. Quantitative inhibition zone area of bacterial colonies formed by (**B**) *S. aureus* and (**D**) *E. coli* with the AgNP-incorporated hydrogels (*n* = 3).

Biomaterials for tissue engineering and regenerative medicine should be free from the risk of infection from pathogenic microorganisms when delivered into a defected or injured area. Thus, antibacterial activity is one of the critical necessities in clinics for the successful treatment and regeneration of damage and injury. The first model, an injectable hydrogel (GC/CMC), was prepared from guanidylated chitosan and carboxymethyl cellulose by self-organization through electrostatic interactions [30]. Since the AgNPs were mixed before gelation (in the sol state of hydrogels), there were no optical changes after the incorporation of AgNPs into the hydrogels. The evaluation of antibacterial activity was performed with *S. aureus* (Gram-positive) and *E. coli* (Gram-negative) bacteria as model microorganisms (Figure 6). An agar petri dish cultured with both microorganisms was placed with the AgNP-incorporated hydrogels and incubated at 37 °C for a day to determine the inhibition zones (Figure 6A,C). The bacterial strains on the plate were totally covered when no gels had been placed. The AgNP-free hydrogel showed no antibacterial effect, and the AgNP-incorporated hydrogels generated inhibition zones, which were extended with increasing AgNP concentrations (Figure 6B,D).

**Figure 7:**
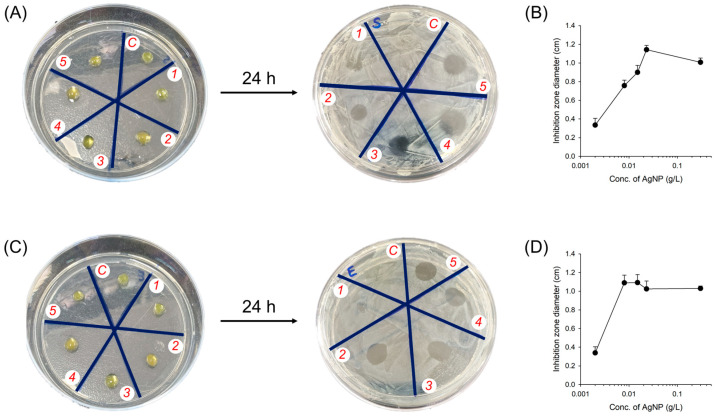
Bactericidal activity of AgNP-incorporated GelMA hydrogels. Representative images of bacterial colonies formed by (**A**) *S. aureus* and (**C**) *E. coli* with the AgNP-incorporated hydrogels were placed on the agar petri dish for a day to analyze the inhibition zone. C = control, AgNP-free hydrogels. Areas 1–5 = AgNP-incorporated hydrogels at concentrations of 2, 8, 15, 23, and 30 mg L^−1^ AgNPs, respectively. Quantitative inhibition zone area of bacterial colonies formed by (**B**) *S. aureus* and (**D**) *E. coli* with the AgNP-incorporated hydrogels (*n* = 3).

Next, we further confirmed the antibacterial activity of AgNPs with a photocrosslinkable hydrogel, GelMA, which is a frequently used hydrogel platform as a second model hydrogel [31,32,33]. After the bacterial strains on the agar plates were covered, the AgNP-incorporated GelMA hydrogels were put on the plate (Figure 7). The occurrence of an inhibition zone was observed after incubation for 24 h at 37 °C (Figure 7A,C). The hydrogels were completely absorbed into the culture medium and remained in the inhibition area after incubation. The AgNP-incorporated hydrogel successfully produced an inhibition zone despite an extremely low concentration of AgNP (2 mg L^−1^). The size of the inhibition zone was gradually extended as the concentration of AgNP increased (Figure 7B,D). These results with both hydrogels suggest that bacterial growth was significantly inhibited by the effect of the synthesized AgNPs. The mechanism of antibacterial activities before and after incorporation into the hydrogel can be explained by silver ions and catecholamine groups released from the AgNPs [3,29]. AgNPs can continually release silver ions, which adhere to the cell walls and cytoplasmic membrane. The adhered ions increase the permeability of the membrane and result in the disruption of the bacterial envelope. The uptake of free silver ions into cells then generates reactive oxygen (ROS) species that disrupt the cell membrane and DNA replication due to the interaction of silver ions with the sulfur and phosphorus groups in DNA. Consequently, AgNPs result in damage to DNA replication and cell reproduction and finally cause the termination of bacteria (Figure 4, Figure 6 and Figure 7) [34].

We also determined a series of characterizations for AgNP-composite GelMA hydrogels, including swelling ratio (Figure S3A), water content (Figure S3B), degradation (Figure S3C), and strain-dependent rheology (Figure S3D). The results showed that there are no significant differences between GelMA with or without AgNPs, suggesting that the incorporation of AgNPs does not significantly affect the physicochemical characteristics of the hydrogels.

### 2.5. Biocompatibility of AgNP-Incorporated Hydrogel Composites

**Figure 8:**
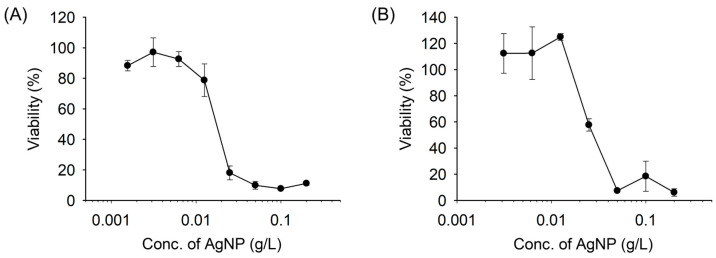
In vitro (**A**) 2D and (**B**) 3D cell viability assay of BMSCs incubated with AgNPs and AgNPs-incorporated hydrogels for 24 h (*n* = 3). The value in the 3D cell viability assay was normalized by the blank hydrogel group.

The biocompatibility of AgNPs was determined in 2D and 3D cell cultures and analyzed using the CCK-8 assay. There was no significant cytotoxicity at concentrations up to approximately 0.01 g/L, supporting the notion that AgNPs are biocompatible with cells (Figure 8). In particular, when the cells were incubated in the 3D AgNP-incorporated hydrogels, the viability of the BMSCs was increased. These results strongly support that the lower cytotoxicity of AgNPs in the hydrogels is because of the limited interaction between the cells and AgNPs in the hydrogel network. In other words, the cell viability demonstrated that the mechanism of AgNP cytotoxicity in 3D hydrogel results from the interaction of positively charged moieties of AgNPs within a hydrogel network [35]. The combinative use with negatively charged hydrogels, such as hyaluronan-based hydrogels, would benefit biosafety improvements for future clinical uses. With predefined results, these results indicate that the AgNP-incorporated hydrogels are highly biocompatible, antibacterial, and antioxidant, resulting in their ability to support cells. Additionally, based on the antibacterial, antioxidant, and cytotoxic potentials, the recommended optimum concentration of AgNPs in the hydrogel is around 0.02 g/L.

## 3. Conclusions

In summary, functional hydrogels with green-synthesized AgNPs were developed by the self-assembly of dopamine as a reducing and antioxidant agent with silver ions. The facile and green-synthesized AgNPs demonstrated irregular nanoscale shapes (mostly spherical) with a positive charge, as determined by DLS measurements and SEM observation. The particles are stable in an aqueous solution for up to 2 weeks. In addition, antibacterial activities against Gram-positive and Gram-negative bacterial strains and antioxidant capabilities were evaluated against DPPH radicals. The GC/CMC and GelMA hydrogels with AgNPs showed a powerful antibacterial effect with good cytocompatibility. This study has shown that the biocompatible hydrogel has antibacterial and antioxidant activities by introducing facile and green-synthesized AgNPs as a potential tool for protection against pathogenic microorganism infections.

## 4. Materials and Methods

### 4.1. Synthesis of AgNPs

AgNPs were prepared by reducing silver ions with dopamine. A round bottom flask was filled with 50 mL of DW, 0.5 mL (0.3 M for AgNP-1 and 0.5 M for AgNP-2) of dopamine hydrochloride, and 0.5 mL (0.6 M for AgNP-1 and 1.0 M for AgNP-2) of silver nitrate. The whole solution was vigorously stirred for an hour at pH 8.0–8.5. The solution was dialyzed with a dialysis membrane (molecular weight cut-off; MWCO = 12,000–14,000; Labotec. Co., Ltd., Seoul, South Korea) in DW for 2 h. Then, the solution was freeze-dried for 3 days. The final product was stored at under −20 °C for further use.

### 4.2. Characterization of AgNPs

The UV–visible absorption spectra of AgNPs were recorded by a multimode microplate reader (BioTek, Inc., Winooski, VT, USA) for 2 weeks. The field emission-scanning electronic microscopy (FE-SEM) images of AgNPs were taken using a JSM-6700F (JEOL, Ltd., Tokyo, Japan). The size and ζ-potential of AgNPs were measured using an ELSZ-2000ZS (Otsuka Electronic Co., Osaka, Japan) with a set of automatic sampling times and analysis at room temperature.

### 4.3. Antibacterial Activity of AgNPs

A culture of Gram-positive bacteria, *Staphylococcus aureus* (*S. aureus*), and Gram-negative bacteria, *Escherichia coli* (*E. coli*) were prepared by suspending a single colony from a lysogen broth (LB) agar culture in 5 mL of sterile LB medium. The counts of bacteria were evaluated by measuring the absorbance of the medium at 600 nm (OD600) using a UV–vis spectrophotometer. The value of OD600 was used to obtain the number of bacterial colony-forming units (CFU) mL^−1^.

The determination of bacterial inhibition zones was performed using the disc diffusion method [1]. A 100 µL sample with a bacterial solution of 10^6^ CFU mL^−1^ was spread on an LB agar plate, AgNP-absorbed paper discs were dipped directly in the AgNP solution and were placed on the plate, and the plate was incubated at 37 °C for 24 h. To assess the hydrogels, 100 µL of AgNP-containing hydrogels were placed on the bacterial-covered plates.

A MIC test using a broth dilution method was conducted against both bacterial strains. Bacterial suspensions (3 mL) in LB broth media were incubated at 37 °C for 24 h. Subsequently, 100 µL of the solution was transferred to a 96-well plate and absorbance was obtained at 600 nm using a multimode microplate reader (BioTek, Inc., Winooski, VT, USA).

### 4.4. Antioxidant Activity of AgNPs

The DPPH radical scavenging activity of AgNPs was assessed. A 0.1 mM solution of DPPH was prepared in ethanol (95%). The DPPH solution (1 mL) was added to 150 µL of AgNPs solution and then incubated in the dark with gentle shaking for 30 min. The activity was assessed by measuring the absorbance at 516 nm.

### 4.5. Preparation of Hydrogels

For the synthesis of guanidylated chitosan (GC), chitosan (2% *w/v*) was dissolved in 100 mL of 1% HCl. Then, 1.06 g of dicyandiamide was added, and the solution was stirred for 2 h at 90 °C. The resulting solution was precipitated in cold ethyl alcohol (EtOH), filtered, and washed several times with fresh cold EtOH. The final precipitates were dried at 60 °C for 24 h. NMR spectra were recorded in deuterium oxide with a drop of deuterium chloride at room temperature using a Bruker NMR Spectrometer at 500 MHz (Figure S1). For the preparation of the GC/carboxymethyl cellulose (CMC) hydrogel, the 3% CMC solution in DW was added dropwise into the 3% GC solution in DW under constant stirring. The ratio of GC and CMC for the hydrogel was 1:1. AgNP was mixed with the GC/CMC hydrogels to obtain the AgNP-incorporated hydrogels.

The synthesis of gelatin methacryloyl (GelMA) was conducted with the previous preparation method [36]. In brief, gelatin (10 g) was dissolved in 100 mL of pH 7.4 phosphate-buffered saline (PBS) at 50 °C. MA (5 mL) was added to the solution and stirred at 50 °C for 2 days. Then, the resulting solution was dialyzed with a dialysis membrane (MWCO = 12,000–14,000; Labotec. Co., Ltd.) in DW at 50 °C for 2 days. The final solution was freeze-dried and stored at −4 °C until further use. NMR spectra were recorded in deuterium oxide using a NMR Spectrometer (Bruker, Billerica, MA, USA) at 500 MHz (Figure S2). Visible-light-mediated GelMA hydrogel was prepared by the previously published methods with modifications [37,38,39,40]. GelMA hydrogels in the presence or absence of AgNPs were prepared by the irradiation of GelMA prepolymer and a riboflavin (6 μM) initiator (200:1; volume ratio) under visible blue light (400–500 nm, 300 mW cm^−2^) for 2 min.

The hydrogels were incubated in PBS for 24 h and freeze-dried. The water content was evaluated using the following Equation (1):

(1) $$\mathrm{Water}\;\mathrm{content}\;\left(\%\right)=\left(\frac{W_{w}-W_{d}}{W_{w}}\right)\times 100,$$

where W_w_ and W_d_ indicate the weight of wet and dry GelMA hydrogels, respectively.

The swelling ratio of the hydrogel was calculated using the following Equation (2):

(2) $$\mathrm{Swelling}\;\mathrm{ratio}\;\left(\%\right)=\left(\frac{W_{s}}{W_{d}}\right)\times 100,$$

where W_s_ and W_d_ indicate the swollen and dry weights of GelMA hydrogels, respectively.

The degradation of hydrogel was calculated over a period of 4 weeks. The hydrogel was incubated in PBS (0.01 M, pH 7.4) at 37 °C. The medium was replaced every 7 days. The weight of the hydrogel was measured after lyophilization.

(3) $$\mathrm{Residual}\;\mathrm{weight}\;\left(\%\right)=\left(\frac{W_{0}}{W_{t}}\right)\times 100,$$

where W_0_ and W_t_ indicate the weights of GelMA hydrogels at time 0 and t, respectively.

### 4.6. Cell Culture and Cell Viability

The mouse BMSC line (D1 cell, CRL-12424) was obtained from American Type Culture Collection (ATCC, Manassas, VA, USA) and cultured in Dulbecco’s Modified Eagle’s Medium (DMEM) with 10% fetal bovine serum (FBS) and 1% penicillin/streptomycin at 37 °C in 5% CO_2_. DMEM, FBS, penicillin, and streptomycin were obtained from Gibco (Manassas, VA, USA).

Cell viability was measured using a CCK-8 assay (GlpBio). Briefly, BMSCs were cultured in 96-well plates with 4 × 10^3^ cells per well and then incubated overnight at 37 °C in 5% CO_2_. AgNPs were added to each well in 100 μL of medium, and the plates were returned to the incubator for 24 h. The cells were washed with PBS and incubated for 3 h with a 10% CCK-8-containing medium. The CCK-8 absorbance was measured at 450 nm using a microplate reader.

For 3D assays, the BMSCs were encapsulated in nanocomposite hydrogels with a concentration of 1 × 10^6^ cells mL^−1^. The BMSCs encapsulated in hydrogels were cultured in culture media at 37 °C in 5% CO_2_. To assess the viability of BMSCs, the hydrogels were washed with PBS and then incubated for 3 h with a 10% CCK-8-containing medium. The CCK-8 absorbance was measured at 450 nm.

### 4.7. Statistical Analysis

Data are presented as mean ± standard deviation (SD) for all results.

## Data Availability

The data that support the findings of this study are available from the corresponding author upon reasonable request.

## Supplementary Materials

The following supporting information can be downloaded at: https://www.mdpi.com/article/10.3390/gels9030183/s1, Figure S1: ^1^H-NMR spectra of guanidylated chitosan; Figure S2: ^1^H-NMR spectra of gelatin and gelatin methacrylate (GelMA); Figure S3: swelling ratios of GelMA and GelMA with AgNPs, the water content of GelMA and GelMA with AgNPs, the residual weight of GelMA and GelMA with AgNPs in PBS at 37 °C for 4 weeks, and strain-dependent (ω = 10 rad s^−1^, 25 °C) rheology of GelMA and GelMA with AgNPs.
