# Supplementary material for: Silver-Composited Polydopamine Nanoparticles: Antibacterial and Antioxidant Potential in Nanocomposite Hydrogels
Source: Gels. 2023 Feb 27;9(3):183. doi: 10.3390/gels9030183 (PMC10048004; doi:10.3390/gels9030183)
Supplement: Supplementary file 1 [file gels-09-00183-s001.zip › gels-2240462-supplementary.pdf]

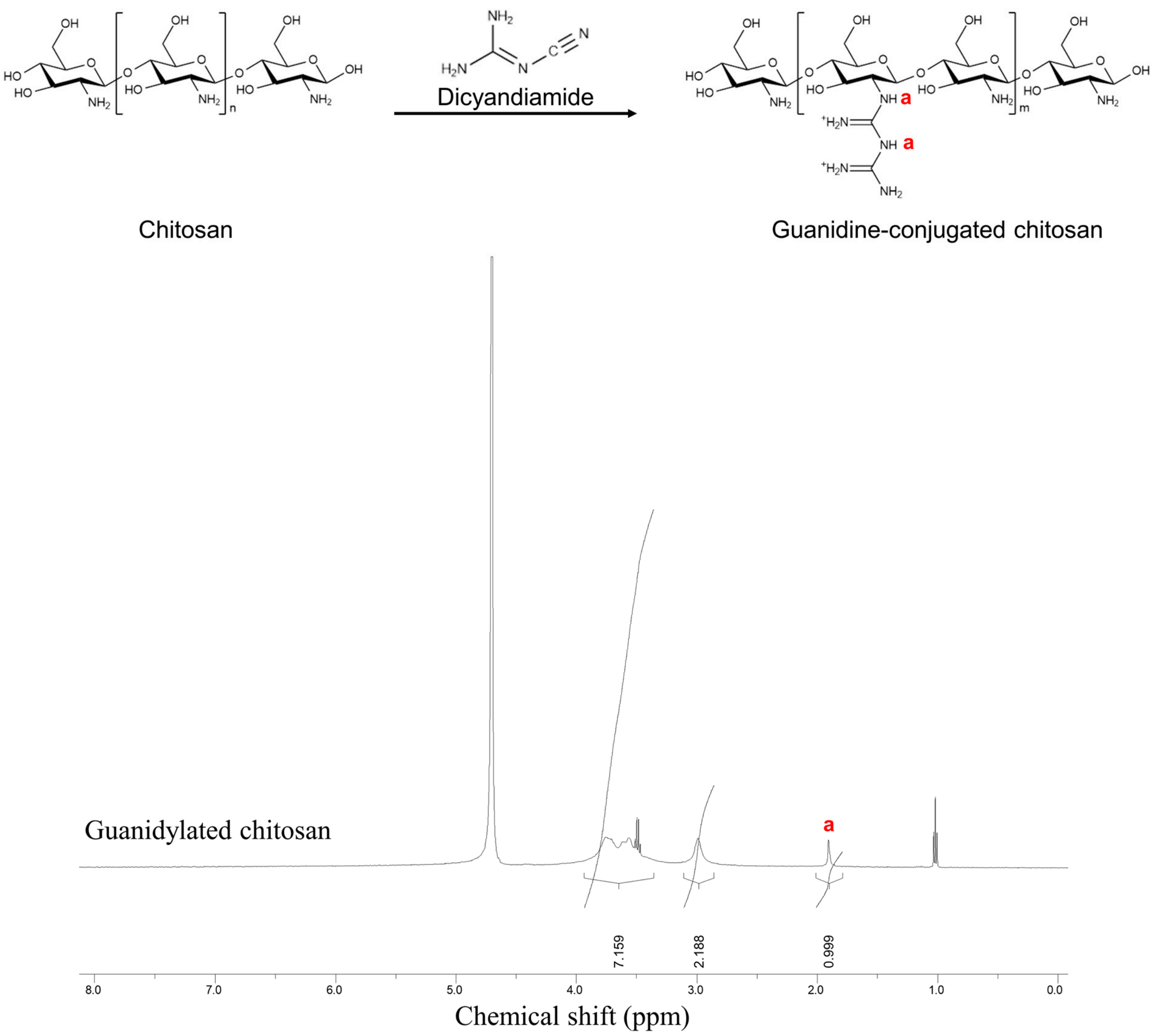

Figure S1. ^{1}H-NMR spectra of guanidylated chitosan. The letter "a" indicates the proton of guanidine-conjugated chitosan.

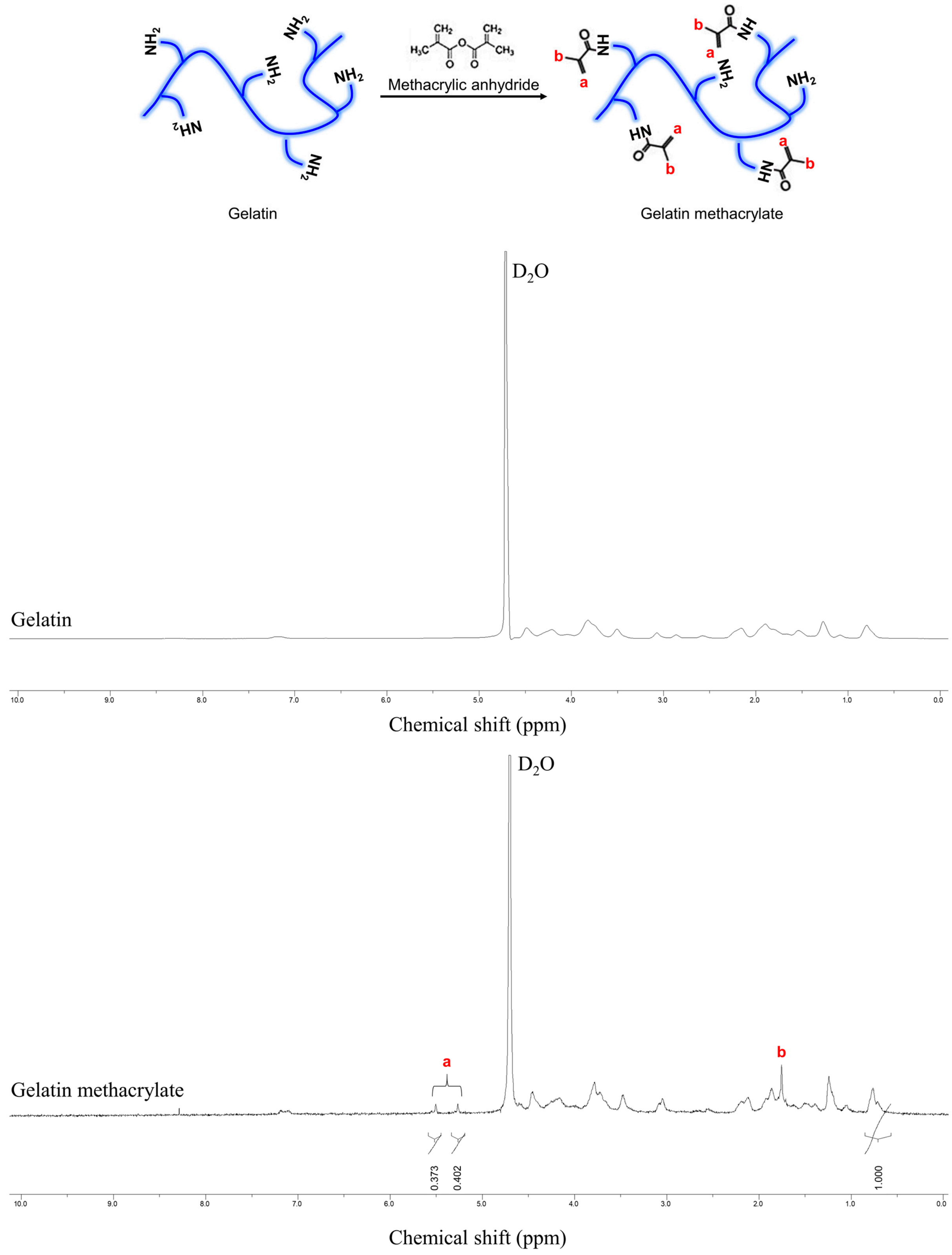

Figure S2. ^{1}H-NMR spectra of gelatin and gelatin methacrylate (GelMA). The letter "a" and “b” indicate the protons of gelatin methacrylate.

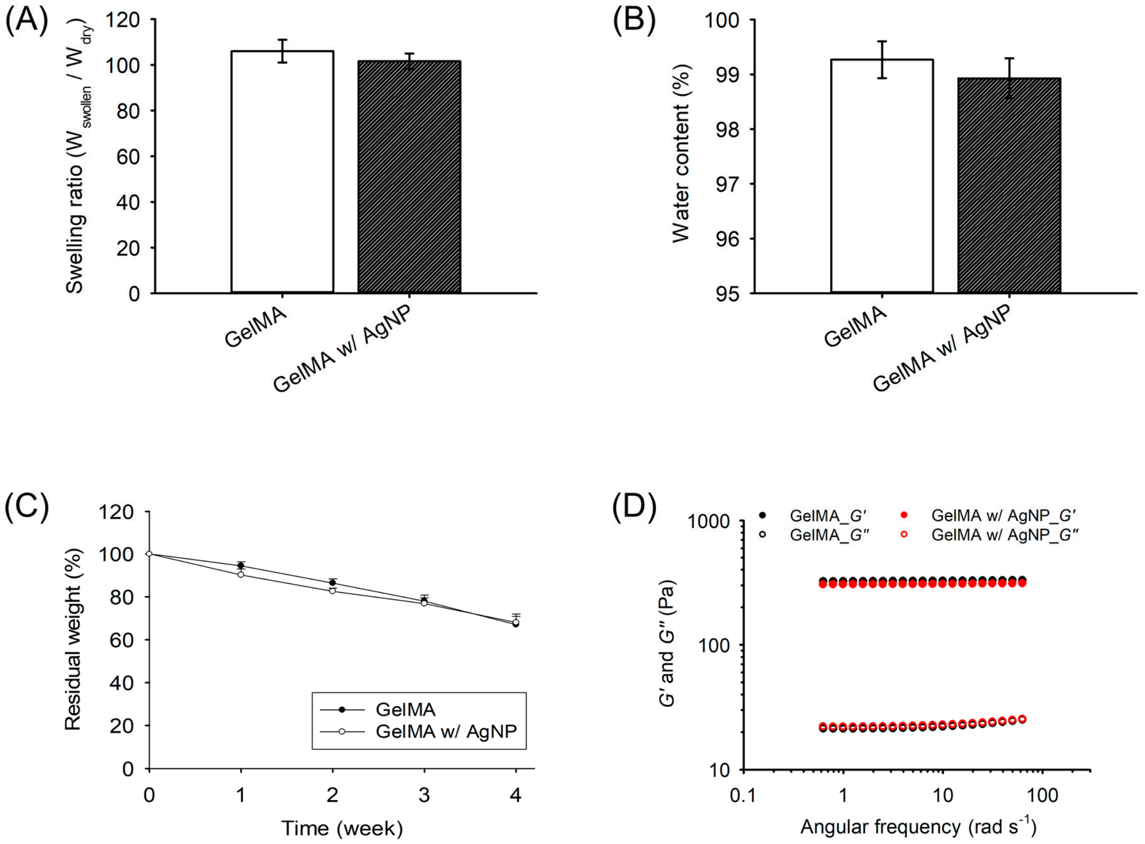

Figure S3. (**A**) Swelling ratios of GelMA and GelMA with AgNPs. (**B**) Water content of GelMA and GelMA with AgNPs. (**C**) Residual weight of GelMA and GelMA with AgNPs in PBS at 37 °C for 4 weeks. Error bars indicate standard deviation (n = 3). (**D**) Strain-dependent (ω = 10 rad s^{-1}, 25 °C) rheology of GelMA and GelMA with AgNPs. The concentration of AgNPs is 30 mg L^{-1}.
